# The Role of Attention in Word Recognition: Results from OB1‐Reader

**DOI:** 10.1111/cogs.12846

**Published:** 2020-06-20

**Authors:** Martijn Meeter, Yousri Marzouki, Arthur E. Avramiea, Joshua Snell, Jonathan Grainger

**Affiliations:** ^1^ LEARN! Research Institute Vrije Universiteit Amsterdam; ^2^ Department of Social Sciences Qatar University; ^3^ Department of Cognitive Psychology Vrije Universiteit Amsterdam; ^4^ Laboratoire de Psychologie Cognitive Centre National de Recherche Scientifique, Aix‐Marseille Université

**Keywords:** Word recognition, Attention, Reading, Computational modeling

## Abstract

When reading, orthographic information is extracted not only from the word the reader is looking at, but also from adjacent words in the parafovea. Here we examined, using the recently introduced OB1‐reader computational model, how orthographic information can be processed in parallel across multiple words and how orthographic information can be integrated across time and space. Although OB1‐reader is a model of text reading, here we used it to simulate single‐word recognition experiments in which parallel processing has been shown to play a role by manipulating the surrounding context in flanker and priming paradigms. In flanker paradigms, observers recognize a central word flanked by other letter strings located left and right of the target and separated from the target by a space. The model successfully accounts for the finding that such flankers can aid word recognition when they contain bigrams of the target word, independent of where those flankers are in the visual field. In priming experiments, in which the target word is preceded by a masked prime, the model accounts for the finding that priming occurs independent of whether the prime and target word are in the same location or not. Crucial to these successes is the key role that spatial attention plays within OB1‐reader, as it allows the model to receive visual input from multiple locations in parallel, while limiting the kinds of errors that can potentially occur under such spatial pooling of orthographic information.

## 1 Introduction

When reading, orthographic information is extracted not only from the word the reader is looking at, but also from adjacent words in the parafovea (e.g., Angele, Tran, & Rayner, 2013; Hohenstein, Laubrock, & Kliegl, 2010; Kennedy, 2000; Snell, Vitu, & Grainger, 2017; Vitu, Brysbaert, & Lancelin, 2004). In the present work we examine, using a recently published computational model (Snell, van Leipsig, Grainger, & Meeter, 2018), how orthographic information can be processed in parallel across multiple words, and in particular the key role of spatial attention in such processing.

Several lines of research have suggested that information from the fovea and the parafovea is processed in parallel. Most prominently, studies of reading have found benefits from previewing words that have not yet been fixated, relative to when those words are masked. This has consistently been the case for the word immediately to the right of the fixated word (Hohenstein et al., 2010; Kennedy, 2000; Rayner, 1998), but also for previews of the word one further to the right (the n+2 word; Risse, Hohenstein, Kliegl, & Engbert, 2014). More critically, when a word in the parafovea is orthographically similar to the fixated word, it can also speed up the recognition of the fixated word (Angele et al., 2013; Dare & Shillcock, 2013; Snell et al., 2017; Vitu et al., 2004).

Other research in support for parallel processing of foveal and parafoveal information stems from masked priming studies with flexible prime and target locations. In these studies a target stimulus must be categorized as word or nonword (*lexical decision*). The target is preceded by a masked prime, and typically lexical decision is faster when primes are identical (or orthographically related) to the target word than when they are not (e.g., Forster & Davis, 1984; Forster, Davis, Schoknecht, & Carter, 1987; Grainger & Jacobs, 1993). In most masked priming studies, target and prime stimuli are both presented centrally, where prime and target are likely processed in a serial fashion. In a few studies, however, primes and target words have been presented off fixation. Marzouki and Grainger (2008, exp 2) presented a target at fixation, preceded by a prime that could occur anywhere from 7° to the left or right of fixation. This study was motivated by the idea that a larger distance between prime and target location would reduce integration between the two, and indeed priming was reduced when prime and target were not presented at the same location. Crucially, however, in a later study, Marzouki, Meeter, and Grainger (2013) found that when primes were always presented on fixation and targets could vary in location, priming was actually independent of location. This suggested that information from prime and target was integrated at some location‐invariant level, which might be difficult to reconcile with anything other than parallel processing of information.

However, sentence reading research has suggested that when the fixated word is followed by a high‐frequency word in the parafovea, this does not speed up processing of the currently fixated word compared to when it is followed by a low‐frequency word (Brothers, Hoversten, & Traxler, 2017). This has been interpreted as evidence against parallel processing at the level of words during reading (Brothers et al., 2017; White, Boynton, & Yeatman, 2019). Some have argued that even orthographic processing occurs for only one word at a time, and that orthographic parafoveal‐on‐foveal effect would instead be driven by a pre‐attentive activation of parafoveal feature detectors (e.g., Angele et al., 2013).

Another body of evidence for parallel letter processing across multiple words, which is less susceptible to alternative explanations, is provided by the *flanking letters lexical decision (FLLD)* paradigm. In a lexical decision task, Dare and Shillcock (2013) presented target words and nonwords surrounded by flanking letter pairs. These pairs either consisted of the first and last letters of the word/nonword (e.g., RO–ROCK–CK), or control letters (e.g., LE–ROCK–SH). Lexical decision was faster in the first case than in the second—that is, the word ROCK was recognized faster when it was flanked by RO and CK than when it was flanked by LE and SH. Such a finding could still be explained by a noisy coding representation (e.g., Gomez, Ratcliff, & Perea, 2008) in which, for example, the flanker to the left is mistaken for the first two letters of the word; in the case of a match this would increase its activation. However, Dare and Shillcock additionally found that recognition was equally speeded in a condition in which flankers were swapped, so that the left two letters of the word flanked it on the right side and vice versa (e.g., CK–ROCK–RO). This result suggests that it is the relative order of the letters within the flankers that aided recognition, not their proximity to the word.

Grainger, Mathôt, and Vitu (2014) proposed a “bag‐of‐bigrams” model in order to account for the results reported by Dare and Shillcock (2013). This involved a simple extension of the Grainger and van Heuven (2003) model of orthographic processing such that processing of location‐specific letter identities is performed in parallel across more than one word, and that this information activates a set of location‐invariant open bigrams. In line with this account of Dare and Shillcock’s (2013) results, Grainger et al. (2014) found that swapping the order of letters within flankers (e.g., OR–ROCK–KC) significantly reduced the benefit from related flankers, relative to flankers with intact letter order. This particular finding is difficult to reconcile with a serial processing perspective: Angele et al.’s (2013) account of orthographic parafoveal‐on‐foveal effects, which ascribes a key role to low‐level feature detectors, would predict no effect of within‐flanker letter order. Additionally, in a study that made use of the principle that the pupil responds to the brightness of covertly (i.e., without looking) attended locations, Snell, Mathôt, Mirault, and Grainger (2018) observed that the pupil size was contingent with the brightness of the locations of flanking stimuli only in conditions where these impacted target processing (i.e., when they were presented left and right of the target, but not above and below the target). This further strengthens the conception that orthographic integration effects are driven by parallel processing.

This being said, the bag‐of‐bigrams account proposed by Grainger et al. (2014) would appear to raise insurmountable problems for the reading system when more than one word is presented on the screen. If there was no code linking letters to location, how would the brain be able to assign letters to words? Would the brain not mix up the letters that belong to different words, creating illusory conjunctions between parts of one word and parts of an adjacent word? One answer would be that top‐down expectations from preceding text may guide word recognition during reading. In line with that idea, illusory conjunctions do occur when such top‐down expectations cannot be used, for example when unrelated words are presented together on the screen (Davis & Bowers, 2004; McClelland & Mozer, 1986; Vandendaele, Snell, & Grainger, 2019). However, even in those circumstances they are rare.

In a recent computational model, the OB1‐reader model (OB1‐Reader, Snell, van Leipsig, et al., 2018), we proposed that spatial attention may prevent the excessive occurrence of illusory conjunctions. While attention would be distributed across multiple words in parallel, the distribution is shaped as a gradient that leads to preferential processing of the bigrams contained within the word in focus, which would give that word a leg up in being recognized faster than words that would be formed from illusory conjunctions of multi‐word bigrams. That attention plays a role in reading is an assumption made by several other well‐known models of reading (Engbert, Nuthmann, Richter, & Kliegl, 2005; Reichle, Warren, & McConnell, 2009), but not all (Reilly & Radach, 2003, 2006).

In our previous paper, we showed that OB1‐reader can account for standard findings in the sentence reading literature, and it can also account for effects of overlap between parafoveal words and the fixated word (Angele et al., 2013; Dare & Shillcock, 2013). As such, OB1 offers a more complete theoretical framework than its sentence reading predecessors, such as E‐Z Reader (Reichle et al., 2009), SWIFT (Engbert et al., 2005), and Glenmore (Reilly & Radach, 2006).

Here we provide a further test of OB1’s broad scope, by treading outside the realm of sentence reading and investigating whether OB1‐reader can also account for findings from priming and flanker studies discussed above. We will first present a short overview of OB1‐reader at a conceptual level. We then show how it can explain findings from masked priming and flanker paradigms. Our last simulation will show that, through its attentional window, the model can recognize words despite the fact that these are flanked by other words. It will show that flankers substantially slow recognition when attention is unfocused. Finally, we will discuss our results and the crucial role of attention in our simulations and in reading.

## 2 Computational model

### 2.1 Structure of the model

**Fig. 1:**
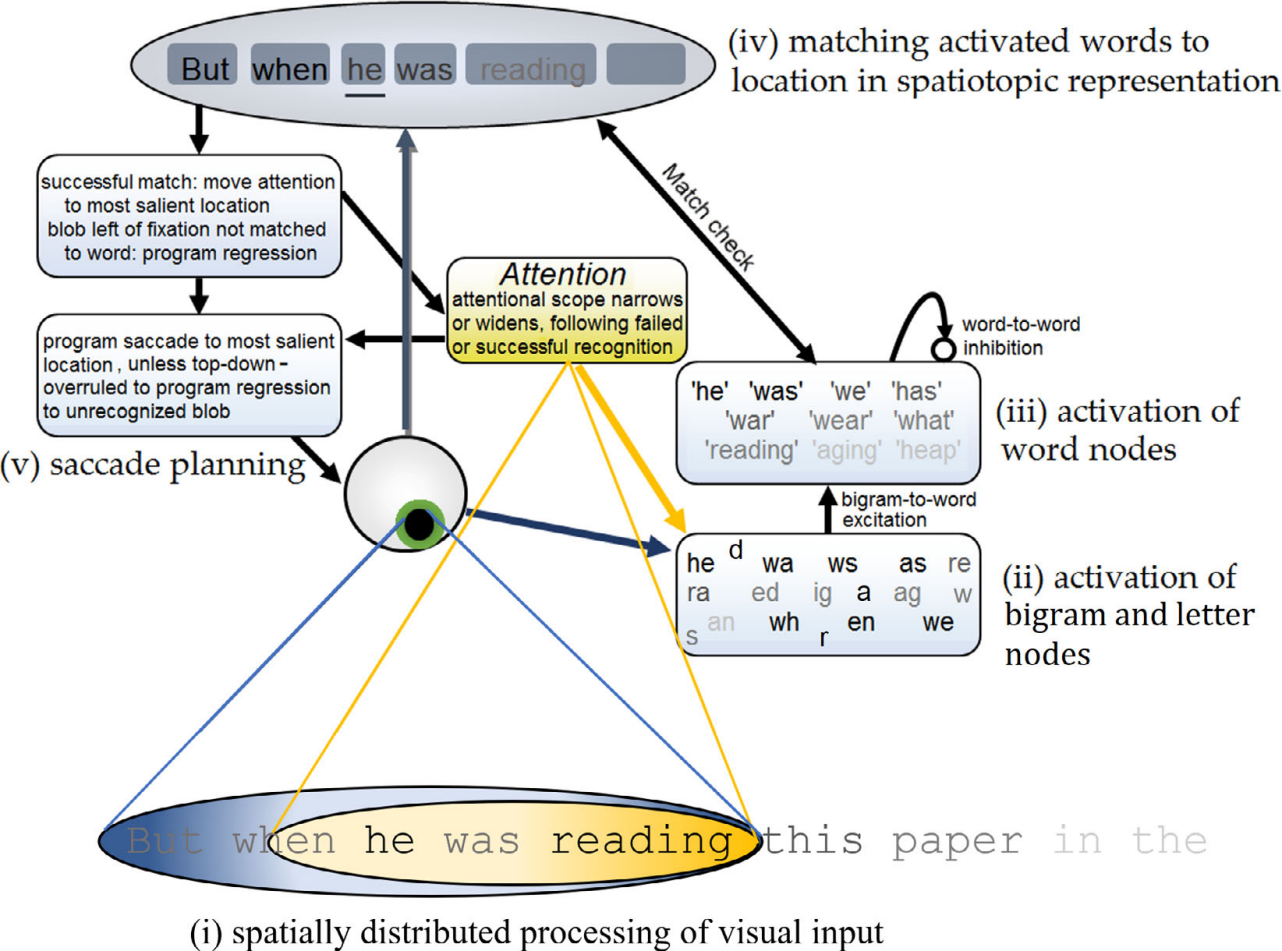
Schematic diagram of OB1‐reader. (i) OB1 sees multiple words at a time. Within the visual input, letter processing is modulated by visual acuity (shown through the contrast of the letters) and visuospatial attention (yellow oval). The attentional window is skewed toward the right. The focus of attention can be shifted independently of the eye’s fixation. (ii) Open‐bigram nodes are activated by the visual input, with stronger activation of letters that are close to the centers of fixation and attention, and stronger activation for letters that suffer less from crowding because they are at a word edge. (iii) Word nodes are activated by open‐bigram nodes coding for the open bigrams that occur in the word (e.g., “reading” by “re” but also “ra”). Word nodes are inhibited by word nodes that share the same bigrams. (iv) Upon fixating a text, OB1 generates a spatiotopic sentence‐level representation that represents expectations about the length of individual words. Word nodes that reach a recognition threshold are matched to locations (“blobs”) in the spatiotopic representation based on length. OB1 only recognizes a word when it can be mapped onto a plausible location. Recognized words generate expectations about upcoming words, through feedback activation of word nodes based on Cloze probability. When a word is successfully recognized, attention moves ahead of the eyes to the most salient location. Each word’s saliency is determined by the proximity of its letters to the centers of fixation and attention. (v) Whether a saccade program is initiated is stochastically determined in each 25 ms processing cycle, with successful word recognition increasing the chance of initiation. The center of the most salient word form in the visual input becomes the saccade target. Saliency‐based saccade targeting is overruled when a word location to the left of fixation has not yet been marked as recognized. Instead, a regression to that location will be executed. The attentional window is widened after each fixation during which a word is successfully recognized, while it is narrowed after each fixation without successful recognition.

OB1‐reader consists of five parts (see Fig. 1). Below, these parts and the organizing principles of the model are introduced, with computational details given in the Appendix.

Visual input to the model is a line of text centered around fixation, with visual acuity and visual attention limiting what information is picked up from this input. To account for crowding effects, letters in the centers of words receive weaker visual input than letters at the edges of words (see Grainger, Dufau, & Ziegler, 2016, for a discussion of the combined effects of crowding and acuity; Grainger, Tydgat, & Isselé, 2010; Perea & Gomez, 2012).

Visual input is assumed to activate a bank of location‐independent letter and open‐bigram units, following Grainger and van Heuven (2003). The letter units code for the presence of single letters, and the open‐bigram units code for the presence of two letters within a letter string in the input in a certain order, though not necessarily in adjacent positions. For example, presentation of the word “READ THIS” activates the bigram unit not only for “RE” and “HI,” but also for “RD” and “HS” (note that “RT” would not be activated because “R” and “T” are in different letter strings). Letter and bigram units receive input from the whole input array, which implies that they are location invariant.

Letter and bigram units in turn activate units coding for words. Activity of word units is updated in discrete 25 ms time steps according to standard interactive activation formulas (McClelland & Rumelhart, 1988). Word units receive excitation from units coding for letters and open bigrams contained within the word (e.g., the unit for “READING” is activated by letter and bigram units R, A, RE, RD, DN, etc.). Word units receive inhibition from other word units, scaled by the number of letters and bigrams that these words share. The assumption underlying this is that inhibitory connections are only formed between words that are often active at the same time, because they share the same inputs. This inhibition helps the model avoid activating multiple words that are plausible interpretations of the same letter string (e.g., “this” and “his” when the input is THIS), but it does not hinder the activation of word units coding for nonoverlapping word (e.g., “read” and “this” when the input is READ THIS).

Visual input also sets up a spatiotopic representation of how many words there are and what their approximate length is. This representation initially has the form of unidentified blobs, but as word nodes are activated, a matching process assigns word identities to blobs that match in length.

The model contains a system to move an attentional window and plan saccades. In reading, the spotlight moves to the visually most salient location (determined by word size, eccentricity, and closeness to the focus of attention) to the right of the current focus of attention, followed by a saccade to the location of the attentional window. However, when the spatiotopic representation contains an unrecognized word to the left of fixation, the spotlight moves to the location of that unrecognized word, and a regression is planned. The width of the attentional window is widened, within a set limit, each time a word is successfully recognized and narrowed if a previous word has not been marked as “recognized.” As such, the attentional distribution is a function of text difficulty and reading proficiency.

### 2.2 New assumptions

In the current work, we used a variant of OB1‐reader (Van Leipsig, 2015) with two additional assumptions not present in the Snell, van Leipsig, et al. (2018) version:

- We assumed that the focus of attention is set at the onset of a word recognition trial so as to encompass all locations where the observer expects the target word to occur. This would be a narrow focus if the observer knows that the word will have a fixed length and will always be at one location (typically at fixation), and a broad focus if the observer is unsure where the word will occur in the visual field, or whether the word will be long or short.
- We assumed that visual onsets result in exogenous shifts of attention toward the location of the onset (Irwin, Colcombe, Kramer, & Hahn, 2000; Theeuwes, 1991; Van der Stigchel et al., 2009), and a subsequent saccade if there is no further visual onset.

These two assumptions determine in the simulations below how attention is steered. For comparison, in the simulations of reading reported in Snell, van Leipsig, et al. (2018), attention either shifts to the visually most salient word to the right when word recognition is proceeding smoothly, or back to an unrecognized word when a word to the left of current fixation was not recognized previously. Those mechanisms are not incompatible with the assumptions made above—they represent different behavior in different situations (visual onsets do not typically occur during reading). Although the exact link between attention and saccades is still debated, it is generally acknowledged that eye movements to a certain location are preceded by a shift of attention to that location (Baldauf & Deubel, 2008). In the case of exogenous saccades—eye movements in response to the onset of a salient stimulus—findings from the transient attention literature suggest that attention is moved to the onset location relatively swiftly, within 50 ms (Nakayama & Mackeben, 1989; Wilschut, Theeuwes, & Olivers, 2011). The eyes then follow with a certain delay, which was set here at 125 ms to reach a typical 175 ms average saccade latency for exogenous saccades (e.g., Meeter & Van der Stigchel, 2013). Processing was then stopped for one 25 ms time step to model saccadic blindness (Erdmann & Dodge, 1898; Vallines & Greenlee, 2006), after which it resumed with an input array centered at the new position of the eyes.

### 2.3 Modeling single‐word recognition paradigms

The model contains quite a number of parameters. However, parameter values for the simulations were taken from van Leipsig (2015), where they were optimized for simulations of text reading. They were thus not changed to optimize the simulations presented here. To simulate paradigms in the single‐word recognition literature, we took a typical setup as the starting point in which all letters were presented in a monospaced font with letter size equal to 0.5 degrees of visual angle. Larger font sizes would lead to somewhat steeper eccentricity effects, but they would not affect the qualitative pattern of results.

For subliminal priming studies, we simulated brief prime exposures by presenting prime words for one time step (i.e., 25 ms) and target words until response, with negligible time in between prime and target word (in the studies that we simulated, a mask was presented for 10 or 12 ms, but this interval was ignored as it was smaller than one time step). We simulated studies in which targets were either consistently presented at fixation while primes were presented at varying locations (Marzouki & Grainger, 2008), or primes were presented at fixation and targets at random locations (Marzouki et al., 2013).

For these simulations we used Dutch words (later simulations were run with English and German words, yielding the same results). The lexicon consisted of the 200 words with highest frequency in Dutch as found in SubtLex_NL_ (Keuleers, Brysbaert, & New, 2010), as well as all words used in an experiment. These could be lists of three‐, five‐, or eight‐letter words, consisting of the highest frequency words with appropriate size in SubtLex_NL_.

Simulation results encompass 200 trials. The simulations were repeated several times, yielding virtually the same results in every replication.

## 3 Results

### 3.1 Flanking letters lexical decision

**Fig. 2:**
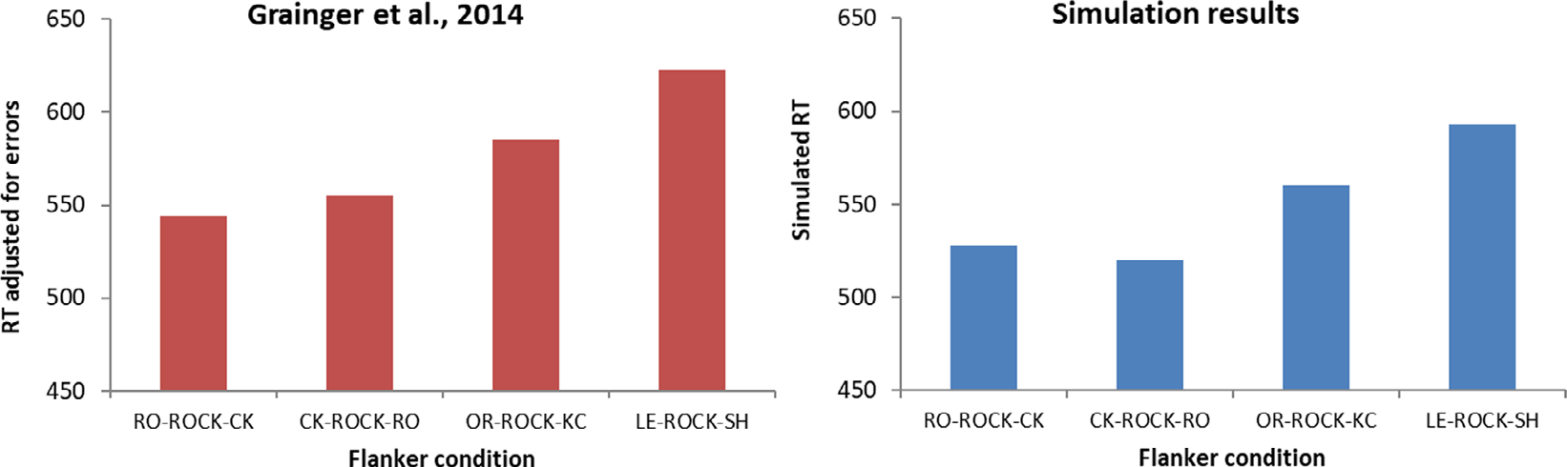
Response times (data) or model word recognition time (simulations) in the flanking letters lexical decision paradigm, in which target words and nonwords were flanked by letter pairs. Only data from the words are shown. Flankers could either be a bigram from the word itself (e.g., RO–ROCK–CK), could be word bigrams in reversed order (CK–ROCK–RO), or reversed bigrams (letter, rev; e.g., OR–ROCK–KC), or could contain letters that are not in the target word (different letters; e.g., LE–ROCK–SH). Empirical data (left panel) from Grainger et al. (2014).

We first simulated the results of Grainger et al. (2014) obtained with the FLLD paradigm. In this version of the lexical decision task, each centrally presented word or nonword target is accompanied by letters placed to the left and to the right and separated from the target by a space. In the Grainger et al. study, flankers could be bigrams contained within the word, or reversed bigrams. As shown in Fig. 2, the model reproduces most findings from the data. Word recognition is faster when flankers consist of bigrams from the same word (e.g., RO–ROCK–CK), relative to when flankers contain different letters than the target word (e.g., LE–ROCK–SH). This is the case whether or not the order of the flankers is reversed (e.g., CK–ROCK–RO). However, the benefit is strongly reduced when the order of the letters within the bigrams is reversed (e.g., OR–ROCK–KC or KC–ROCK–OR, which are not differentiated in Fig. 2 but resulted in similar model recognition times). The key element in the model that allows it to successfully simulate these effects is that bigram nodes are location invariant: They receive input from the whole visual field and thus do not differentiate between a flanker on one side of the word and a flanker on the other side.

Reversed bigrams (OR–ROCK–KC) do result in faster lexical decision than flankers that consist of letters not present in the word (LE–ROCK–SH). The model captures this, too, thanks to nodes within the open‐bigram pool coding for single letters, which do activate the right target word even when bigram order is reversed. There was no difference in simulated reaction time for CK–ROCK–RO compared with RO–ROCK–CK. The slight advantage for RO–ROCK–CK that seems present in the data of Grainger et al. (2014) was in fact not significant, and it was also not present in Dare and Shillcock’s (2013) Experiment 1 with the same paradigm.

### 3.2 Priming as a function of prime and target location

Another set of studies that also seems to suggest parallel processing of input from the whole visual scene is that of Marzouki, Meeter, and Grainger (2008) and Marzouki et al. (2013).

**Fig. 3:**
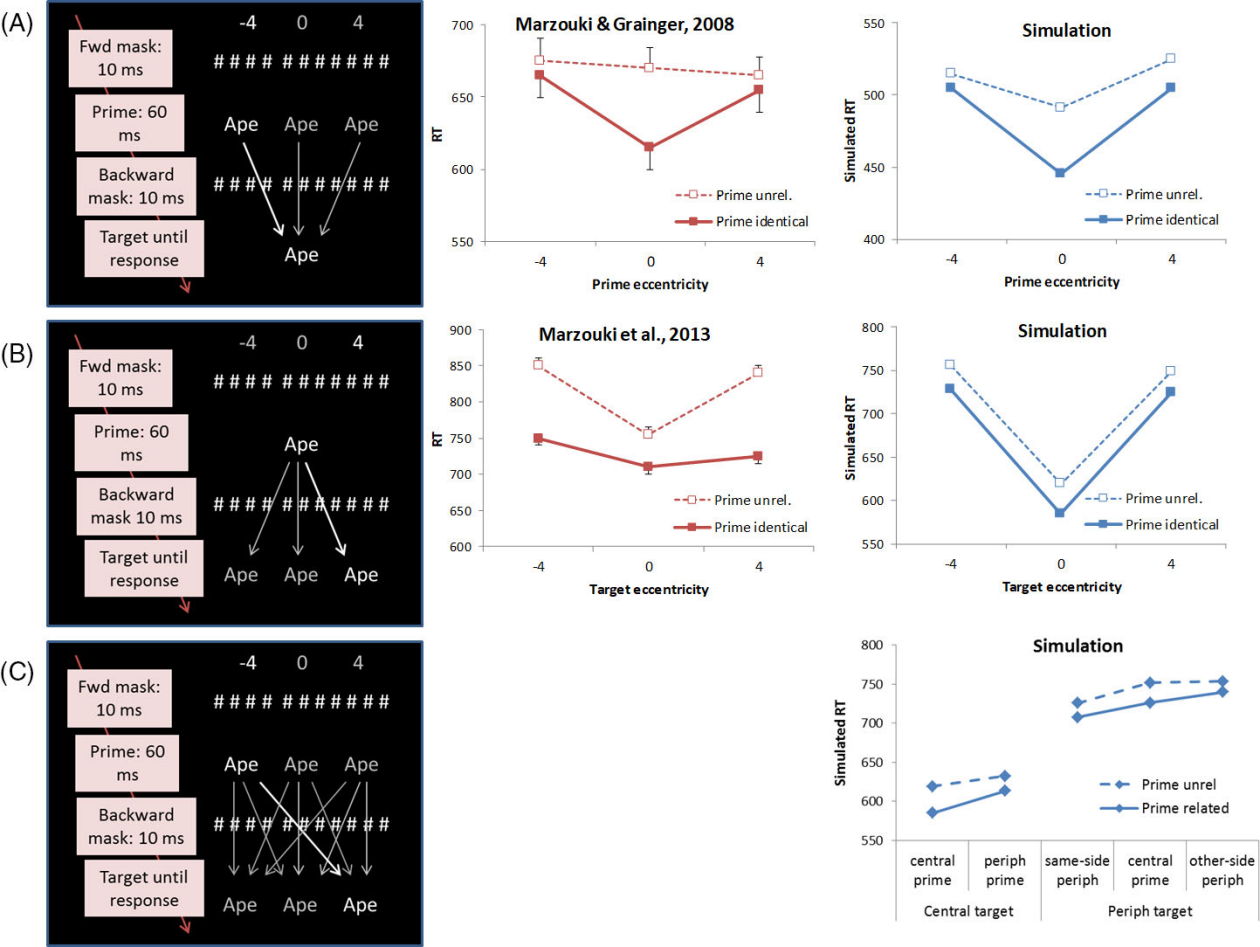
Schematic and results of three masked priming simulations in which prime and/or target location was varied (central vs. peripheral). For two of those, data are presented from lexical decision experiments. Trials in which pseudowords were shown were present in all studies, but these are not shown here. (A) In Experiment 1B of Marzouki and Grainger (2008), primes could appear at various locations, while targets were always presented centrally. Error bars give 1 SEM. (B) In Experiment 2 of Marzouki et al. (2013), primes were always presented at fixation, while the subsequent target word could be presented at fixation (0⁰) or left (−4⁰) or right (4⁰) of it. Error bars give 1 SEM. (C) The full crossover design, in which both primes and targets could appear at multiple locations on the screen, has not been tested yet with word stimuli. Results from the OB1 simulation (right panel) stand as predictions. Shown in all the graphs is lexical decision time for words (A‐B) or simulated word recognition time (A‐C).

In Experiment 1B of Marzouki and Grainger (2008), participants performed lexical decision on three‐letter targets that were always presented at fixation. A prime stimulus preceded the target for 60 ms and was then masked (participants who could detect prime identities with these presentation times were excluded from the analysis of the results). Primes could be presented either at fixation or to the left or right of it. A prime could either be the same word/pseudoword as the later target (“identical” condition) or a word/pseudoword with no orthographic overlap (“unrelated” condition). A priming effect, defined as an RT benefit for targets preceded by an identical versus an unrelated prime, was only evident when target and prime position matched, that is, when both were presented at the center (see Fig. 3A).

As an observer knows where to expect the target word, attention can be narrowly focused around fixation (in line with the assumption outlined in the Computational Model section). To simulate this experiment, we therefore let the model adopt a narrowly focused attention around fixation (with a width equal to the size of the word), at the expected target location. This resulted in central primes being processed and facilitating target processing in the identical condition, but peripheral primes not being processed strongly, and resulting only in very small priming effects (Fig. 3A, right panel).

In Experiment 2 of Marzouki et al. (2013), primes were always presented centrally. Targets could be presented centrally, but also to the left or right of fixation. Participants were slower to respond to peripherally presented targets than to centrally presented targets. However, priming was independent of whether prime and target were presented at the same location (i.e., both centrally), or the prime was presented centrally and the target peripherally—suggesting that input from differing prime and target locations was integrated without penalty.

To simulate this study, we let OB1‐reader start a trial with a broad attentional window (with a width equivalent to 20‐letter spaces)—since it does not know where the target will appear. When the prime is presented, its visual onset directs OB1‐reader’s attention to the central location. This leads to faster responses to central targets (matching the location of attention) than to peripheral targets, which is also seen in the data of Marzouki et al. (2013—see Fig. 3B). A prime thus acts as a spatial cue, capturing attention (also see Marzouki, Grainger, & Theeuwes, 2007). However, such capture effects were independent of the size of priming, which was the same for all three target locations. This can simply be explained by the time course of attentional capture: Although it is fast (Nakayama & Mackeben, 1989; Wilschut et al., 2011), the redeployment of attention still comes *after* the prime has already been replaced by the mask. OB1‐reader thus captures the two main findings of Marzouki et al. (2013), Experiment 2, again thanks to its attentional dynamics and its location‐invariant open‐bigram units.

A more extreme test of location invariance would be if target and prime positions were fully crossed, and right hemifield primes could be shown to prime left hemifield targets and vice versa. Such an experiment was performed by Marzouki et al. (2008), using letter and pseudoletter stimuli instead of words. Again they found that while a prime at the same location as the target (e.g., both at fixation) led to faster responding overall, priming was independent of whether prime and target were presented at the same location. A prime in the opposite hemifield as the target still primed it as efficiently as a prime presented at the same location as the target. We simulated how a similar experiment with word stimuli would be handled by OB1‐reader. The simulations show that primes capturing attention would lead to faster responding when the target appears at the same location as the prime (conditions central prime–central target and same‐side peripheral prime–peripheral target in Fig. 3C). However, the size of priming (difference between “identical” and “unrelated” in Fig. 3C) was not dependent on whether prime and target were presented at the same location. Since this condition has not yet been tested with word stimuli, it stands as a prediction of OB1. However, it matches the results, obtained with letter identification, of Marzouki et al. (2008).

Note that in the simulation there is always a benefit for central targets relative to peripheral ones, independent of prime location. This has two reasons. First, visual acuity is better at fixation than in the periphery, which is incorporated in the model through stronger visual input for the center of fixation. Second, attention is already at the central location at the start of the trial, which means that there is no processing time lost due to attention having to move to a peripheral location. The benefit for central targets, independent of prime location or prime‐target relatedness, was also found by Marzouki et al. (2008).

### 3.3 Effects of attention and word length

The simulations presented above establish that visual attention can help explain findings in the word recognition literature. However, they do not offer a rationale for why reading would depend on attention. Reilly and Radach (2003) have noted that “not much is gained” in explanatory power “by assuming that ‘attention’ is disengaged, moved and re‐allocated…” (p. 432). The theoretical framework adopted in OB1‐reader suggests, on the contrary, that visual attention does play a crucial role in reading, namely by boosting processing of visual input from one word over that from surrounding words. This allows the model to recognize the words in the text as opposed to seeing false conjunctions of letters from different words. Here, we test that rationale by exploring how the model would perform with either a wide attentional window, when presented with respectively an isolated word or a text.

We presented the model with words of 3, 5, 8, or 10 letters that were either presented alone or surrounded by two random words of equal length to approximate the visual input gained during a given fixation in text reading. Attention was either narrowly focused on the central word by setting the width of the attentional window equal to the width of the word, or broadly focused, with a width equal to a 20‐letter position.

**Fig. 4:**
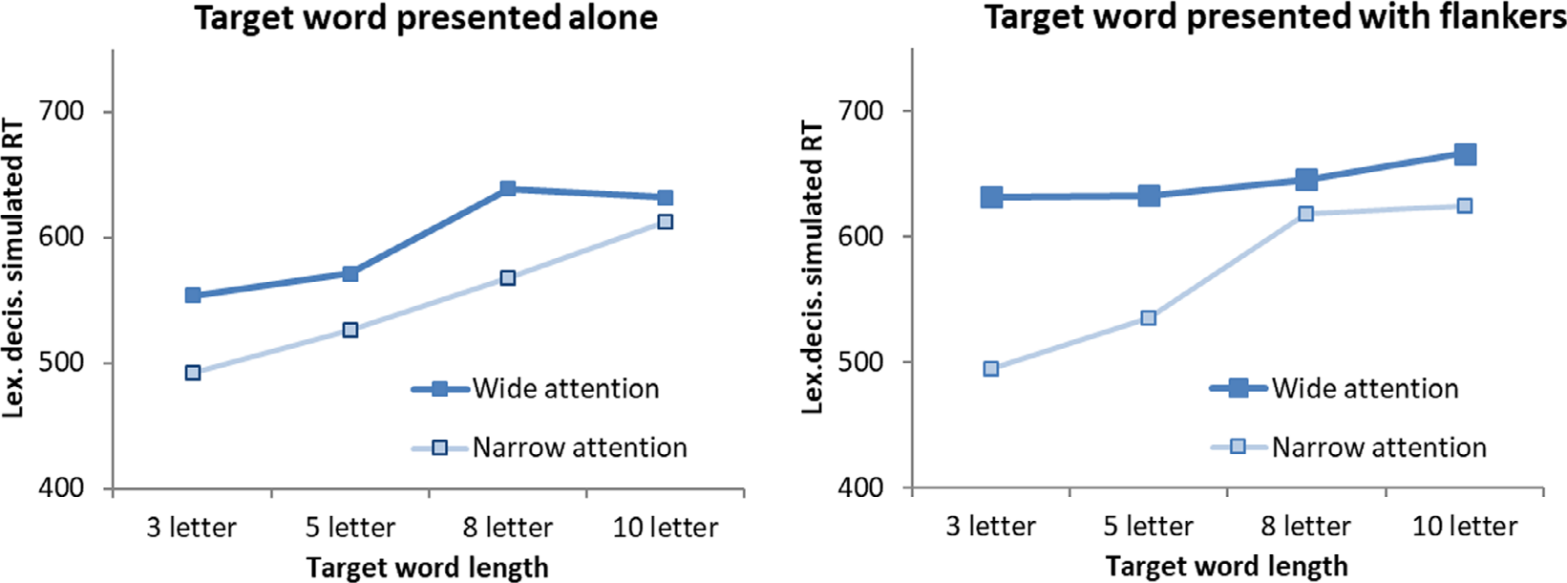
Model lexical decision time as a function of number of letters in the word, whether the word was flanked by other words or not, and whether attention was narrowly focused on the word or not.

Fig. 4 shows the result of these simulations. As is typically found in reading (Kliegl, Grabner, Rolfs, & Engbert, 2004), the model showed a word length effect. This effect was attenuated when the attentional window was wide, which would correspond to a condition in which it is unclear how long the presented word will be. The word length effect was amplified for narrow attention when a word was presented with flankers, as it would be inside a text. Indeed, word length effects are stronger when analyzed in text reading, with fixation duration as primary measure, than in paradigms in which words are presented in isolation, such as lexical decision (New, Ferrand, Pallier, & Brysbaert, 2006). The difference between a narrow and wide attentional window was amplified in the condition with flankers. OB1‐reader also made errors in this condition—for long words when attention is narrow (12% for 10‐letter words, none for shorter words), but also for short words when attention was wide (11% for 10‐letter words, 7% for eight‐letter words, and 1% for five‐letter words). The errors were driven by the fact that amalgams of letters from the target word and flankers led to erroneous recognition. This confirms that OB1‐reader relies on visual attention to identify individual words in text, with the constraints imposed by the gradient guarding against illusory conjunctions between letters that reside in different words.

**Fig. 5:**
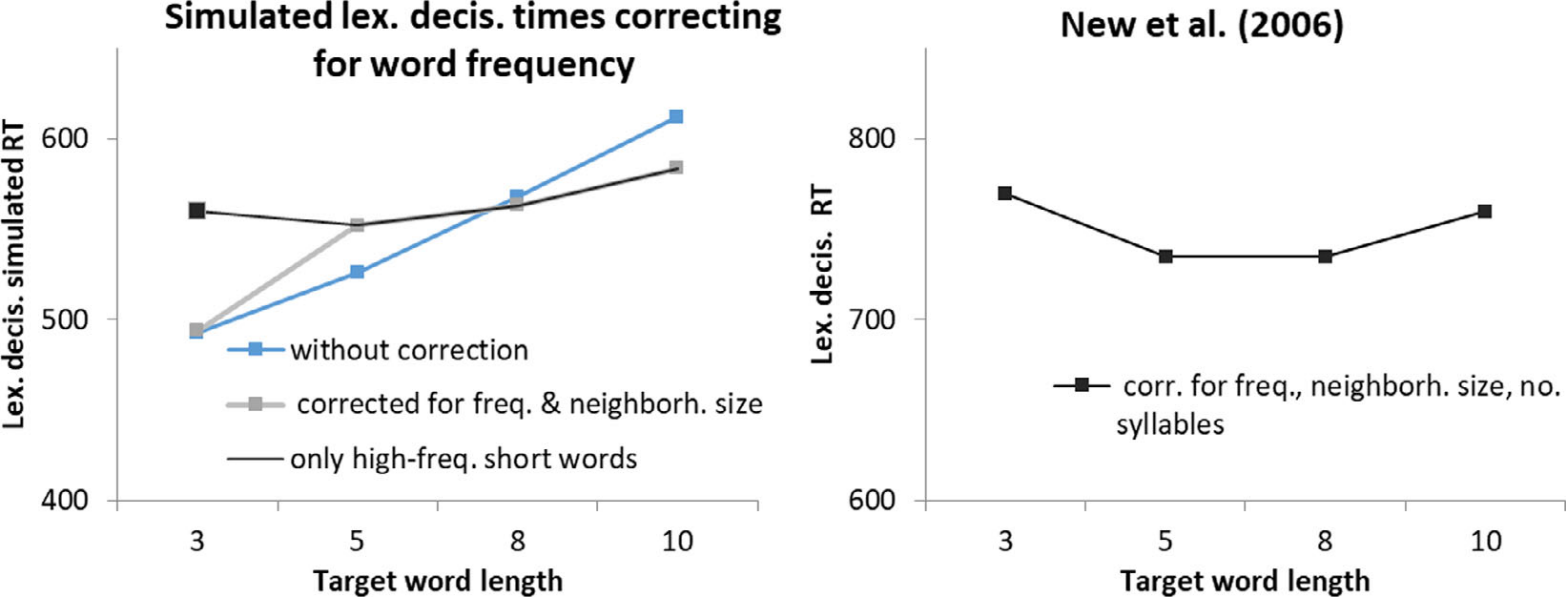
Model lexical decision times in the narrow attention, words presented alone condition of Fig. 4, corrected for word frequency and neighborhood size. For comparison, the results from New et al. (2006) are redrawn. Note that in New et al. lexical decision times were also corrected for number of syllables in words, which was not available for our set.

Lexical decision time typically increases monotonously with word length, but it has been argued that this is a result of word frequency confounds: When reaction times are corrected for word frequency and neighborhood size differences, a different pattern emerges. Short words typically are high frequency, and correcting for this reveals a U function with shortest lexical decision times for words with length between five and eight letters, and longer reaction times for both shorter and longer words (New et al., 2006). We corrected the results in the *narrow attention, words presented alone* condition of Fig. 4 for word frequency (log‐10 frequency per million, taken from SubtLex_NL_, Keuleers et al., 2010) and neighborhood size, using parameters of New et al. (2006). This attenuated the effect of word length but did not yield the U function found by New et al. The stimulus set used in the simulations contained untypically many low‐frequency short words. When we restricted the analysis to the 30 three‐letter words with SubtLex_NL_ frequency above 5000, a weak U function appeared (see Fig. 5), reproducing the result of New et al. (2006). However, the fact that this result was not apparent using all three‐letter words in our simulation shows that the model recognizes short words faster than would be typical of human readers.

## 4 Discussion

Here, we show that OB1‐reader can not only account for many findings in the sentence reading literature (Snell, van Leipsig, et al., 2018), but can also successfully reproduce findings from the FLLD paradigm and from lexical decision with masked primes. It thus reconciles findings from two literatures that are not yet connected very well; namely, those on text reading and on single‐word recognition.

Two assumptions are central to OB1‐reader’s success in accounting for the findings covered in this paper. First, input to the word recognition system is based on relative coding, as incorporated in the open‐bigram scheme in which absolute letter position is not coded. Second, visual attention allows the brain to boost input from one word, allowing it to avoid the mingling of bigrams from different words that would seem a natural consequence of the first assumption. Together, these two assumptions allow the model to account for several findings that would otherwise appear to be puzzling, without the need for any parameter fitting. The model naturally integrates orthographic information from primes and flankers that are spatially disconnected from the target word, since bigrams contribute to word recognition independent of their location. It thus reproduces the finding that the location of a flanker is immaterial to its impact, but that the order of letters within the flankers does affect word recognition.

The model also naturally produces the effect of target word expectation, implicit in the studies of Marzouki et al. (Marzouki & Grainger, 2008; Marzouki et al., 2008, 2013). In these studies, prime location did affect performance when participants knew where the target word would appear, allowing them to focus attention (Marzouki & Grainger, 2008); but prime location had no effect when the target location was unknown, and participants thus had to adopt a wide attentional window (Marzouki et al., 2008, 2013). In addition, the model simulates the fact that prime onset captures attention, and slows or speeds up target recognition depending on whether the target is at the same location as the prime, or not. That visual onsets capture attention is widely accepted in the literature on visual attention (Irwin et al., 2000; Theeuwes, 1991; Van der Stigchel et al., 2009), but it has not yet been incorporated in the literature on reading and word recognition. The same is true for the assumption of an attentional window of variable width (Theeuwes, 1994; Van der Stigchel et al., 2009).

In a third simulation, we confirmed that the incorporation of an attentional window allows the model to read words embedded in a text without difficulty, even though the model contains no mechanism to separate out bigrams belonging to the word from those belonging to other words. As a bonus, the model offers an explanation for the finding that word length has a stronger effect on the time needed to process a word embedded in a text than on the recognition of a word presented in isolation (New et al., 2006). It is only when other words may interfere with the processing of a target word that focusing attention on the target word yields strong benefits.

Hence, the simulations presented here offer an answer to the challenge raised by Reilly and Radach (2003), namely what is gained by assuming that an attentional window operates in reading. The simulations presented here would suggest that attention drives the ability to disentangle parallel processed words.

## Appendix A

The model operates in discrete time steps that are equated to 25 ms. To compare model performance to lexical decision reaction times, a fixed 400 ms was added to the time it took to recognize a word (70 ms visual processing time, 300 ms post‐lexical processing decision time, and 30 ms motor response time).

### 1.1 Input and letter/bigram activation

On each time step, the input to the model consists of an array of letters, with positions of the letters coded relative to fixation. As it is well known that acuity diminishes with eccentricity, visual input *v_i_* generated by a letter *i* was assumed to be a decreasing function of eccentricity *e_i_*., which, for a lack of better estimates, was given as the cortical magnification in V1 (the increase in cortical space devoted to locations closer to fixation). Moreover, visual input was a function of the attentional weight *a_i_* and a masking factor *m_i_*. Within a word, letters mask one another such that they are less visible in the center than at the edges, or than they would be on their own (Marzouki & Grainger, 2014). This is captured by *m_i_*. Together, these three factors determined *v_i_* in the following way:

(1) $$v_{i}=a_{i}*m_{i}\left[1/c_{e}\left(0.018*e_{i}+\frac{1}{0.64}\right)\right]$$

The term within round brackets was taken from Harvey and Dumoulin’s (2011) estimate of cortical magnification in humans. Since this formula computes millimeters of cortex, a constant *c_e_* was added that rescaled magnification so that its maximum value, obtained at fixation, was 1 (*c_e_* = 33.7). Equation 5 describes attentional weight *a_i_*, while *m_i_* was set to 2 when *i* was a border letter, and 0.5 when it was a center letter.

Bigram nodes were activated whenever their constituent letters i and j were both present within the same word, and within four letters of one another (i.e., a maximum of three letters in between). Output *O_ij_* of such a bigram node was computed as:

(2) $$O_{\mathrm{ij}}=\sqrt{v_{i}v_{j}}\;\text{if}\;i,\;j\;\text{within four letters and same word},\;0\;\text{otherwise}$$

Activity of a letter node *i* was computed using the same formula, with *i* substituted for *j* (resulting in *v_i_* being squared). If a letter or bigram appeared multiple times within the input array, input to it as defined by equation 2 was simply summated.

### 1.2 Word activation

Activity of a word *w*, *S_w_*, is initialized as 0, and it is bound to the interval between 0 and 1. It is updated on each time step with the following difference equation:

(3) $$\Delta S_{w}=-\tau S_{w}+\left(1-S_{w}\right)*\left[c_{1}\left(\sum_{i,j\in w}O_{\mathrm{ij}}\right)-c_{2}\left(\sum_{!\left(i,j\in w\right)}O_{\mathrm{ij}}\right)-c_{3}\left(\sum_{k}d_{w,k}S_{k}\right)\right]$$

In this equation, the first right‐hand term gives passive decay back to a value of 0 (τ = .08). The second term describes the input to the word node, multiplied by a factor (1‐*S_w_*) that effectuates an asymptotic increase toward the maximum activity (equal to 1). The input consists of, first, excitatory input from bigram and letter nodes that are part of the word. This sum is multiplied by a constant *c*_1_ (equal to 0.01). The second part of the input gives a uniform feed‐forward inhibition from bigram and letter nodes, multiplied by constant *c*_2_ (equal to 0.007). The third part of the input is inhibition from other word nodes. We restricted this inhibition to similar words, on the assumption that the inhibitory connections were formed while both word nodes were active, which will occur more often for words of similar length and overlapping in letters than for dissimilar words. Activity from each word node k is therefore weighted by a factor *d_w,k_* that is equal to the number of letters and bigrams shared between the words, but only if the length of the words is similar (more precisely, that the difference in length between the two words is not larger than 0.25 times the length of the longest of the two plus 1). This term is multiplied by constant *c_3_* (equal to 0.025).

A word *w* is considered recognized when it approximately matches the length of a word in view and activity *S_w_* crosses a threshold T. The first criterion is operationalized as that one of the five words closest to fixation (in a normal text, the fixated word and two on each side of it) contains as many letters as w does, or so that the difference in length between w and the word in the text is not larger than 0.25 times the length of the longest of the two plus 1 (the same criterion as holds for inhibition between word nodes). The threshold *T* for word *w* is set by the following formula:

(4) $$T=\frac{2\ln\left(freq_{\max}\right)-\ln\left(freq_{w}\right)}{2\ln\left(freq_{\max}\right)}*\left(0.04l_{w}\right)$$

Here *l_w_* is the length of a word, which was factored in because long words contain more bigrams than short words, and therefore generate more input to their word node, which can lead to false alarms if the threshold is not adjusted for these words. *Freq_w_* is the frequency with which *w* occurs within a reference corpus (we used SubtlexUS for English and Subtlex‐NL for Dutch (Keuleers et al., 2010)), and *freq_max_* the frequency of the most frequent word within that corpus. With this formula, the threshold is twice as high for a very infrequent word as for the most frequent word in the corpus. This was incorporated under the assumption that frequent words are expected by the brain (Baayen, 2010), and that this lowers the threshold to recognize a word.

### 1.3 Attention

Attention was modeled as a Gaussian with a flexible focus and width. The focus could be the point of fixation, but it could also be on another letter in the display (for practical purposes we scaled attention on a scale of letters, assuming a standard 0.4 letters per visual degree). The attentional weight *a_i_* that a letter *i* received was a function of *f_i_*, its distance, in letters, to the focus of attention, and attentional width:

(5) $$a_{i}=\frac{1}{\text{width}}*e^{-\frac{{\left(f_{i}\right)}^{2}}{2*{\text{width}}^{2}}}+c_{a}$$

This function describes a Gaussian centered around an attention focus, with the standard deviation of the Gaussian functioning as a changeable width. Outside of the Gaussian, the attentional weight was set to constant *c_a_* (set to 0.3).

The following assumptions were made about attentional and eye movement control (with those not made in Snell, van Leipsig, et al. (2018) marked by *):

When a single word is presented on the screen, the focus of attention will be placed at its center and attentional width is set so that two times the width covers the word (i.e., so that the word border is at one SD from the focus of attention).*

When a participant expect a word of a certain length at a certain location, they will fixate at the center of the expected word location and also attend there; attentional width is also set so that two times the width covers the word.*

Each visual onset leads to a shift of the focus attention to its location, which occurs 50 ms after the onset (Nakayama & Mackeben, 1989). This is important in priming experiments in which prime and target can occur at multiple locations and are unexpected visual onsets.*

Each attentional shift leads to a reflexive saccade to the attended location after 125 ms, unless there is another attentional shift.*

Each eye movement leads to 25 ms of saccadic blindness, in which no processing takes place.
